# Treatment of Prostatic Abscess: Role of Transrectal Ultrasound Guided Needle Aspiration

**DOI:** 10.5812/numonthly.2665

**Published:** 2012-03-01

**Authors:** Timur H. Kuru, Boris Hadaschik

**Affiliations:** 1 Department of Urology, Heidelberg University Hospital, University of Heidelberg, Heidelberg, Germany

**Keywords:** Ultrasound, Perinum, Transrectal, Prostate

Dear Editor,

The report by Yadav et al. concerned 12 patients with abscesses within the prostate which were treated with transrectal ultrasound (TRUS) guided needle aspiration (1). Of these 12 patients, 4 had residual abscesses detected in a follow-up TRUS after 1 week and 3 needed further intervention. All patients were treated with antibiotics for 4 weeks.

The treatment of acute prostatitis has frequently been described (2-5). There is wide consensus, that abscesses of the prostate which are visible in a TRUS should be evacuated (4). If the abscess is located in the transition zone of the prostate or concomitant benign prostatic hyperplasia is present, a transurethral resection of the prostatic tissue can also be performed (2). Abscess drainage can be accomplished using either aspiration alone or by temporary drainage placement. This decision should be based on the patient’s general condition, age, comorbidities and abscess volume. If such an intervention is needed, we would recommend the transperineal approach either for needle aspiration or drainage placement. This type of technique has been described previously (6).

The advantage of the perineal approach is in preserving the integrity of the rectal wall and Denonvillier’s fascia to avoid further periprostatic or systemic spread of bacteria during the intervention, which could lead to general sepsis.

Regarding the residual abscess rate of 33% (4/12) intermittent drainage instead of pure aspiration may have been a more appropriate treatment approach. However, this decision has to be made with consideration of all the patient’s clinical aspects at the time of admittance. Regardless of the intervention a 4-week antibiotic treatment according to the microbiology results is common standard practice.
